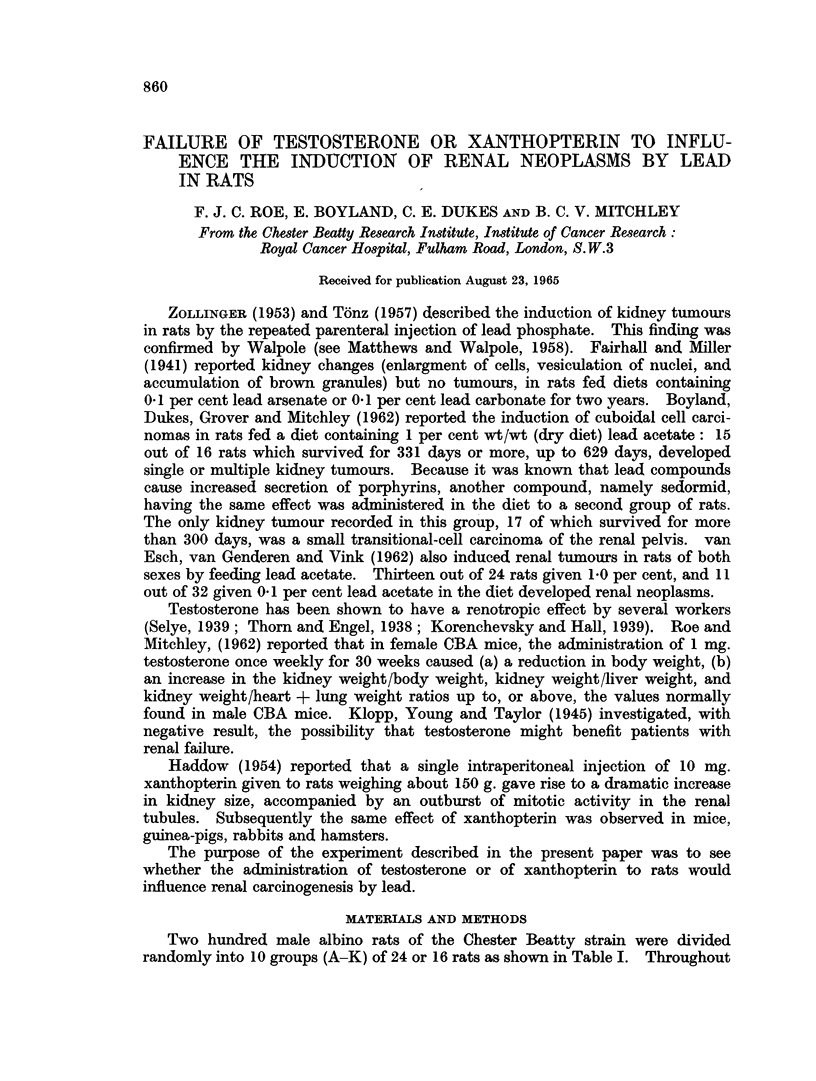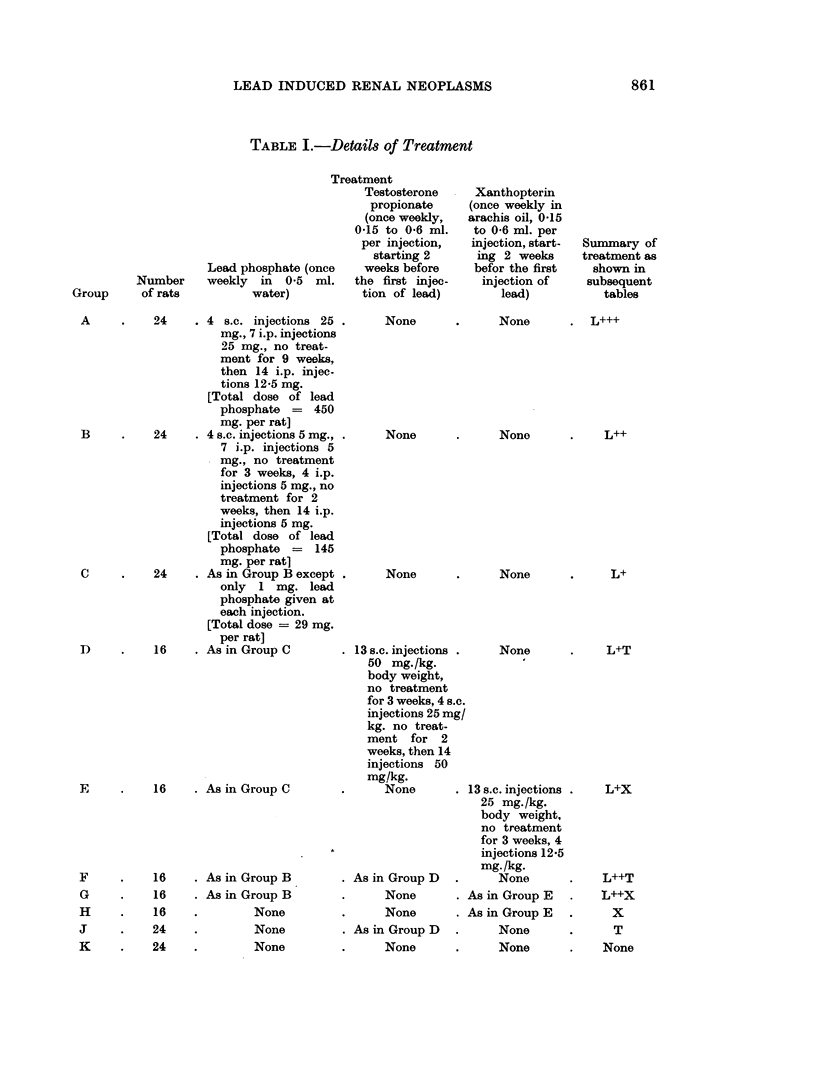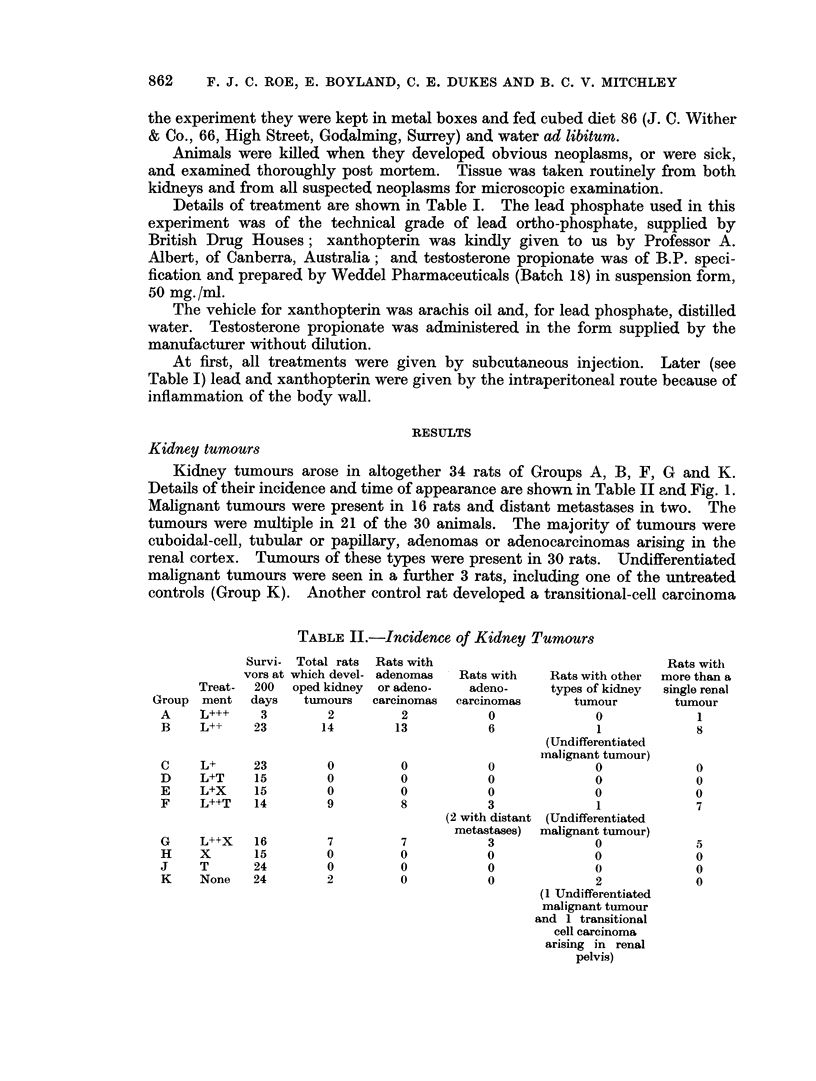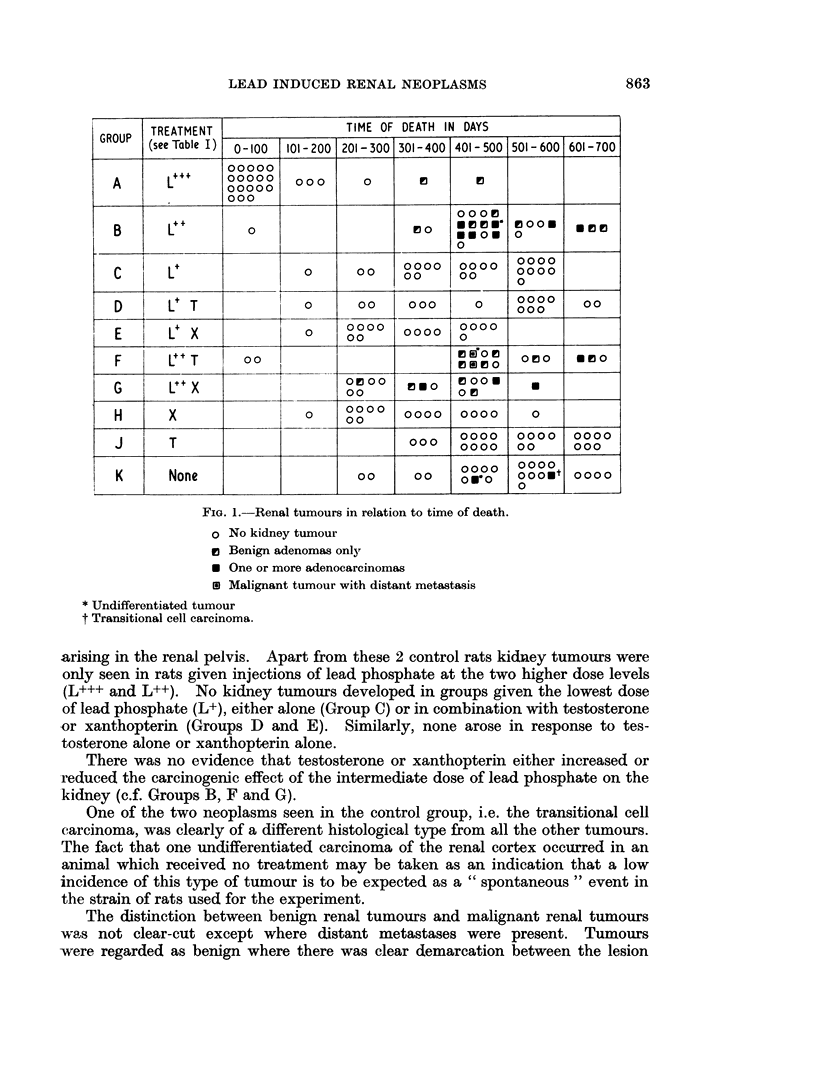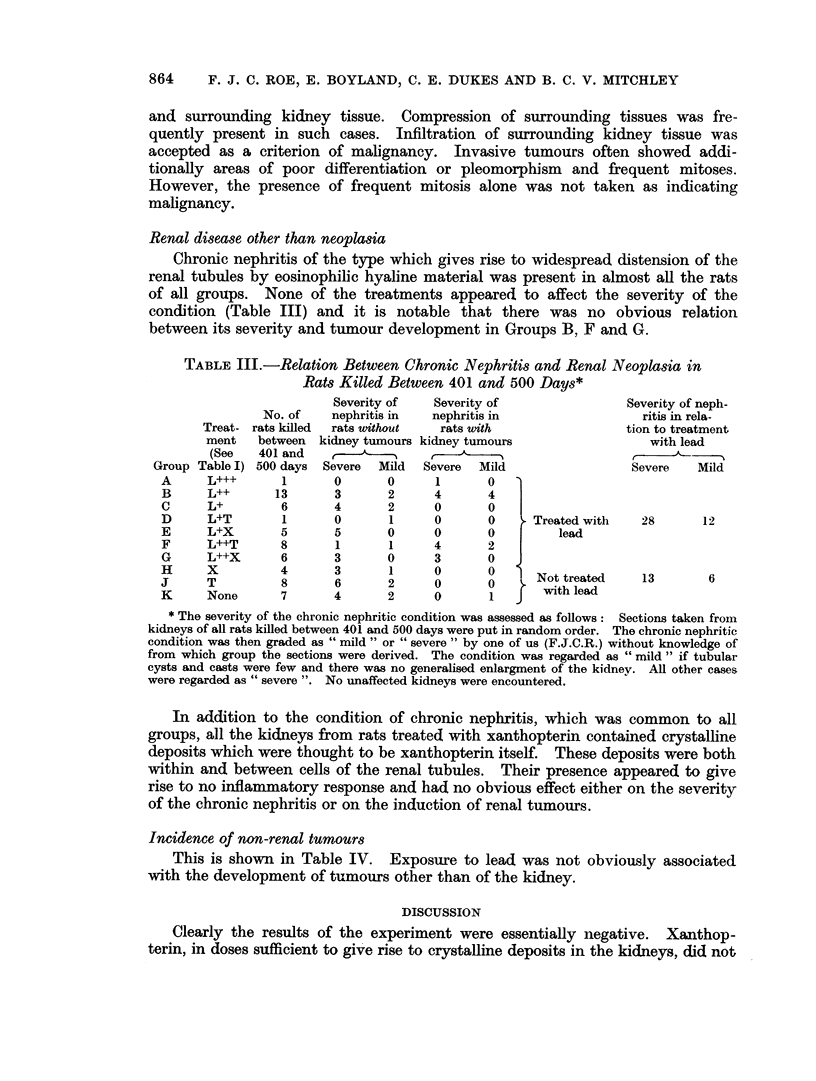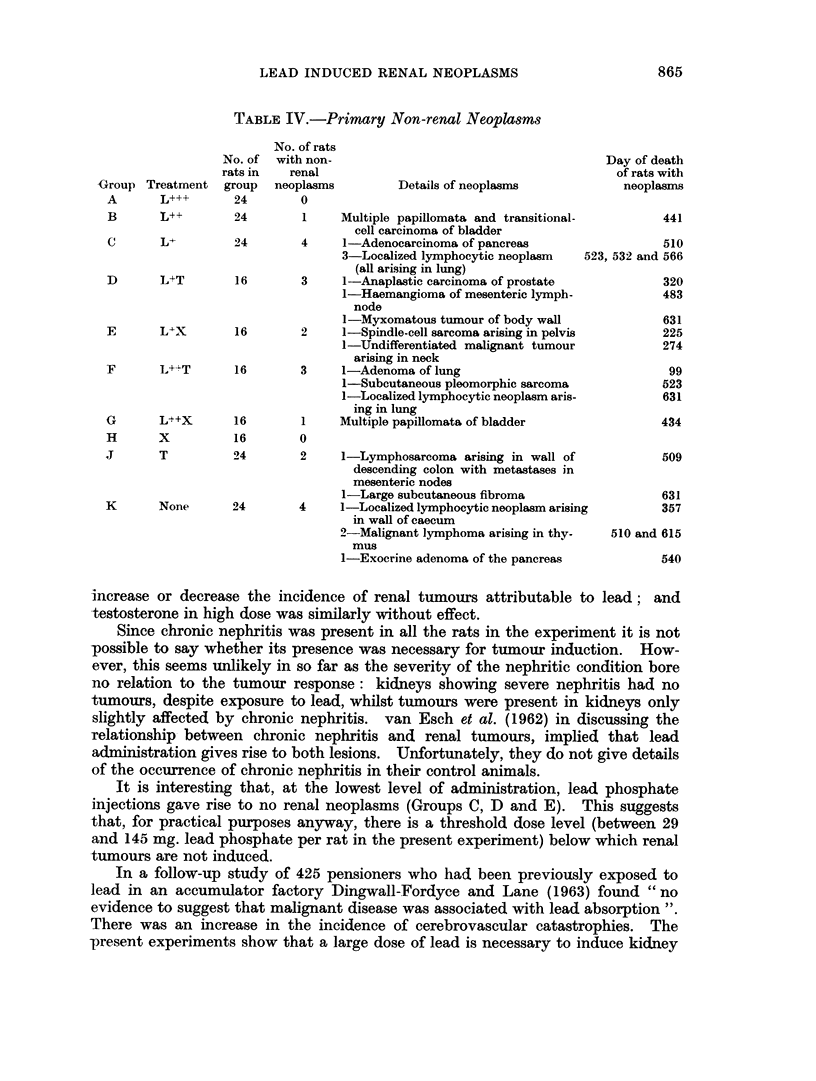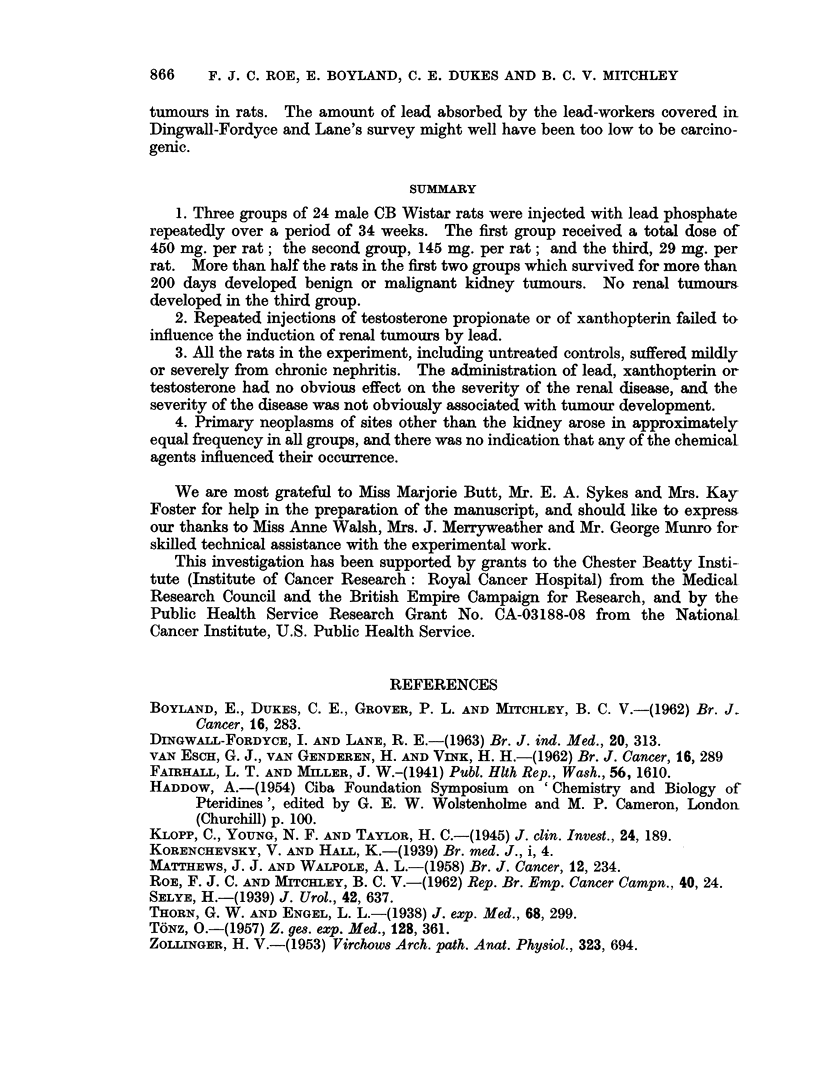# Failure of testosterone or xanthopterin to influence the induction of renal neoplasms by lead in rats.

**DOI:** 10.1038/bjc.1965.99

**Published:** 1965-12

**Authors:** F. J. Roe, E. Boyland, C. E. Dukes, B. C. Mitchley


### FAILURE OF TESTOSTERONE OR XANTHOPTERIN TO INFLUENCE THE INDUCTION OF RENAL NEOPLASMS BY LEAD IN RATS

F. J. C. ROE, E. BOYLAND, C. E. DUKES AND B. C. V. MITCHLEY

*From the Chester Beatty Research Institute, Institute of Cancer Research : Royal Cancer Hospital, Fulham Road, London, S.W.3*

Received for publication August 23, 1965

ZOLLINGER (1953) and Tönz (1957) described the induction of kidney tumours in rats by the repeated parenteral injection of lead phosphate. This finding was confirmed by Walpole (see Matthews and Walpole, 1958). Fairhall and Miller (1941) reported kidney changes (enlargment of cells, vesiculation of nuclei, and accumulation of brown granules) but no tumours, in rats fed diets containing 0·1 per cent lead arsenate or 0·1 per cent lead carbonate for two years. Boyland, Dukes, Grover and Mitchley (1962) reported the induction of cuboidal cell carcinomas in rats fed a diet containing 1 per cent wt/wt (dry diet) lead acetate : 15 out of 16 rats which survived for 331 days or more, up to 629 days, developed single or multiple kidney tumours. Because it was known that lead compounds cause increased secretion of porphyrins, another compound, namely sedormid, having the same effect was administered in the diet to a second group of rats. The only kidney tumour recorded in this group, 17 of which survived for more than 300 days, was a small transitional-cell carcinoma of the renal pelvis. van Esch, van Genderen and Vink (1962) also induced renal tumours in rats of both sexes by feeding lead acetate. Thirteen out of 24 rats given 1·0 per cent, and 11 out of 32 given 0·1 per cent lead acetate in the diet developed renal neoplasms.

Testosterone has been shown to have a renotropic effect by several workers (Selye, 1939 ; Thorn and Engel, 1938 ; Korenchevsky and Hall, 1939). Roe and Mitchley, (1962) reported that in female CBA mice, the administration of 1 mg. testosterone once weekly for 30 weeks caused (a) a reduction in body weight, (b) an increase in the kidney weight/body weight, kidney weight/liver weight, and kidney weight/heart + lung weight ratios up to, or above, the values normally found in male CBA mice. Klopp, Young and Taylor (1945) investigated, with negative result, the possibility that testosterone might benefit patients with renal failure.

Haddow (1954) reported that a single intraperitoneal injection of 10 mg. xanthopterin given to rats weighing about 150 g. gave rise to a dramatic increase in kidney size, accompanied by an outburst of mitotic activity in the renal tubules. Subsequently the same effect of xanthopterin was observed in mice, guinea-pigs, rabbits and hamsters.

The purpose of the experiment described in the present paper was to see whether the administration of testosterone or of xanthopterin to rats would influence renal carcinogenesis by lead.

### MATERIALS AND METHODS

Two hundred male albino rats of the Chester Beatty strain were divided randomly into 10 groups (A–K) of 24 or 16 rats as shown in Table I. Throughout

TABLE I.—*Details of Treatment*

| Group | Number of rats | Treatment: Lead phosphate (once weekly in 0·5 ml. water) | Testosterone propionate (once weekly, 0·15 to 0·6 ml. per injection, starting 2 weeks before the first injection of lead) | Xanthopterin (once weekly in arachis oil, 0·15 to 0·6 ml. per injection, starting 2 weeks befor the first injection of lead) | Summary of treatment as shown in subsequent tables |
|---|---|---|---|---|---|
| A | 24 | 4 s.c. injections 25 mg., 7 i.p. injections 25 mg., no treatment for 9 weeks, then 14 i.p. injections 12·5 mg. [Total dose of lead phosphate = 450 mg. per rat] | None | None | $L^{+++}$ |
| B | 24 | 4 s.c. injections 5 mg., 7 i.p. injections 5 mg., no treatment for 3 weeks, 4 i.p. injections 5 mg., no treatment for 2 weeks, then 14 i.p. injections 5 mg. [Total dose of lead phosphate = 145 mg. per rat] | None | None | $L^{++}$ |
| C | 24 | As in Group B except only 1 mg. lead phosphate given at each injection. [Total dose = 29 mg. per rat] | None | None | $L^{+}$ |
| D | 16 | As in Group C | 13 s.c. injections 50 mg./kg. body weight, no treatment for 3 weeks, 4 s.c. injections 25 mg/kg. no treatment for 2 weeks, then 14 injections 50 mg/kg. | None | $L^{+}T$ |
| E | 16 | As in Group C | None | 13 s.c. injections 25 mg./kg. body weight, no treatment for 3 weeks, 4 injections 12·5 mg./kg. | $L^{+}X$ |
| F | 16 | As in Group B | As in Group D | None | $L^{++}T$ |
| G | 16 | As in Group B | None | As in Group E | $L^{++}X$ |
| H | 16 | None | None | As in Group E | X |
| J | 24 | None | As in Group D | None | T |
| K | 24 | None | None | None | None |

the experiment they were kept in metal boxes and fed cubed diet 86 (J. C. Wither & Co., 66, High Street, Godalming, Surrey) and water *ad libitum*.

Animals were killed when they developed obvious neoplasms, or were sick, and examined thoroughly post mortem. Tissue was taken routinely from both kidneys and from all suspected neoplasms for microscopic examination.

Details of treatment are shown in Table I. The lead phosphate used in this experiment was of the technical grade of lead ortho-phosphate, supplied by British Drug Houses; xanthopterin was kindly given to us by Professor A. Albert, of Canberra, Australia; and testosterone propionate was of B.P. specification and prepared by Weddel Pharmaceuticals (Batch 18) in suspension form, 50 mg./ml.

The vehicle for xanthopterin was arachis oil and, for lead phosphate, distilled water. Testosterone propionate was administered in the form supplied by the manufacturer without dilution.

At first, all treatments were given by subcutaneous injection. Later (see Table I) lead and xanthopterin were given by the intraperitoneal route because of inflammation of the body wall.

### RESULTS

### *Kidney tumours*

Kidney tumours arose in altogether 34 rats of Groups A, B, F, G and K. Details of their incidence and time of appearance are shown in Table II and Fig. 1. Malignant tumours were present in 16 rats and distant metastases in two. The tumours were multiple in 21 of the 30 animals. The majority of tumours were cuboidal-cell, tubular or papillary, adenomas or adenocarcinomas arising in the renal cortex. Tumours of these types were present in 30 rats. Undifferentiated malignant tumours were seen in a further 3 rats, including one of the untreated controls (Group K). Another control rat developed a transitional-cell carcinoma

TABLE II.—*Incidence of Kidney Tumours*

| Group | Treatment | Survivors at 200 days | Total rats which developed kidney tumours | Rats with adenomas or adenocarcinomas | Rats with adenocarcinomas | Rats with other types of kidney tumour | Rats with more than a single renal tumour |
|---|---|---|---|---|---|---|---|
| A | $L^{+++}$ | 3 | 2 | 2 | 0 | 0 | 1 |
| B | $L^{++}$ | 23 | 14 | 13 | 6 | 1 (Undifferentiated malignant tumour) | 8 |
| C | $L^{+}$ | 23 | 0 | 0 | 0 | 0 | 0 |
| D | $L^{+}T$ | 15 | 0 | 0 | 0 | 0 | 0 |
| E | $L^{+}X$ | 15 | 0 | 0 | 0 | 0 | 0 |
| F | $L^{++}T$ | 14 | 9 | 8 | 3 (2 with distant metastases) | 1 (Undifferentiated malignant tumour) | 7 |
| G | $L^{++}X$ | 16 | 7 | 7 | 3 | 0 | 5 |
| H | X | 15 | 0 | 0 | 0 | 0 | 0 |
| J | T | 24 | 0 | 0 | 0 | 0 | 0 |
| K | None | 24 | 2 | 0 | 0 | 2 (1 Undifferentiated malignant tumour and 1 transitional cell carcinoma arising in renal pelvis) | 0 |

| GROUP | TREATMENT (see Table I) | TIME OF DEATH IN DAYS | | | | | | |
| --- | --- | --- | --- | --- | --- | --- | --- | --- |
| | | 0-100 | 101-200 | 201-300 | 301-400 | 401-500 | 501-600 | 601-700 |
| A | $L^{+++}$ | ooooo<br>ooooo<br>ooooo<br>ooo | ooo | o | ◩ | ◩ | | |
| B | $L^{++}$ | o | | | ◩o | ooo◩<br>■◩◩■*<br>■■o■<br>o | ◩oo■<br>o | ■◩◩ |
| C | $L^{+}$ | | o | oo | oooo<br>oo | oooo<br>oo | oooo<br>oooo<br>o | |
| D | $L^{+}$ T | | o | oo | ooo | o | oooo<br>ooo | oo |
| E | $L^{+}$ X | | o | oooo<br>oo | oooo | oooo<br>o | | |
| F | $L^{++}$ T | oo | | | ◩▣*o◩<br>◩▣◩o | o◩o | ■◩o |
| G | $L^{++}$ X | | | o◩oo<br>oo | ◩■o | ◩oo■<br>o◩ | ■ | |
| H | X | | o | oooo<br>oo | oooo | oooo | o | |
| J | T | | | | ooo | oooo<br>oooo | oooo<br>oo | oooo<br>ooo |
| K | None | | | oo | oo | oooo<br>o■*o | oooo<br>ooo■†<br>o | oooo |

FIG. 1.—Renal tumours in relation to time of death.

o No kidney tumour
◩ Benign adenomas only
■ One or more adenocarcinomas
▣ Malignant tumour with distant metastasis

* Undifferentiated tumour
† Transitional cell carcinoma.

arising in the renal pelvis. Apart from these 2 control rats kidney tumours were only seen in rats given injections of lead phosphate at the two higher dose levels ($L^{+++}$ and $L^{++}$). No kidney tumours developed in groups given the lowest dose of lead phosphate ($L^{+}$), either alone (Group C) or in combination with testosterone or xanthopterin (Groups D and E). Similarly, none arose in response to testosterone alone or xanthopterin alone.

There was no evidence that testosterone or xanthopterin either increased or reduced the carcinogenic effect of the intermediate dose of lead phosphate on the kidney (c.f. Groups B, F and G).

One of the two neoplasms seen in the control group, i.e. the transitional cell carcinoma, was clearly of a different histological type from all the other tumours. The fact that one undifferentiated carcinoma of the renal cortex occurred in an animal which received no treatment may be taken as an indication that a low incidence of this type of tumour is to be expected as a " spontaneous " event in the strain of rats used for the experiment.

The distinction between benign renal tumours and malignant renal tumours was not clear-cut except where distant metastases were present. Tumours were regarded as benign where there was clear demarcation between the lesion and surrounding kidney tissue. Compression of surrounding tissues was frequently present in such cases. Infiltration of surrounding kidney tissue was accepted as a criterion of malignancy. Invasive tumours often showed additionally areas of poor differentiation or pleomorphism and frequent mitoses. However, the presence of frequent mitosis alone was not taken as indicating malignancy.

### *Renal disease other than neoplasia*

Chronic nephritis of the type which gives rise to widespread distension of the renal tubules by eosinophilic hyaline material was present in almost all the rats of all groups. None of the treatments appeared to affect the severity of the condition (Table III) and it is notable that there was no obvious relation between its severity and tumour development in Groups B, F and G.

TABLE III.—*Relation Between Chronic Nephritis and Renal Neoplasia in Rats Killed Between* 401 *and* 500 *Days**

| Group | Treatment (See Table I) | No. of rats killed between 401 and 500 days | Severity of nephritis in rats *without* kidney tumours: Severe | Mild | Severity of nephritis in rats *with* kidney tumours: Severe | Mild | | Severity of nephritis in relation to treatment with lead: Severe | Mild |
|---|---|---|---|---|---|---|---|---|---|
| A | $L^{+++}$ | 1 | 0 | 0 | 1 | 0 | | | |
| B | $L^{++}$ | 13 | 3 | 2 | 4 | 4 | | | |
| C | $L^{+}$ | 6 | 4 | 2 | 0 | 0 | | | |
| D | $L^{+}T$ | 1 | 0 | 1 | 0 | 0 | Treated with lead | 28 | 12 |
| E | $L^{+}X$ | 5 | 5 | 0 | 0 | 0 | | | |
| F | $L^{++}T$ | 8 | 1 | 1 | 4 | 2 | | | |
| G | $L^{++}X$ | 6 | 3 | 0 | 3 | 0 | | | |
| H | X | 4 | 3 | 1 | 0 | 0 | | | |
| J | T | 8 | 6 | 2 | 0 | 0 | Not treated with lead | 13 | 6 |
| K | None | 7 | 4 | 2 | 0 | 1 | | | |

* The severity of the chronic nephritic condition was assessed as follows : Sections taken from kidneys of all rats killed between 401 and 500 days were put in random order. The chronic nephritic condition was then graded as " mild " or " severe " by one of us (F.J.C.R.) without knowledge of from which group the sections were derived. The condition was regarded as " mild " if tubular cysts and casts were few and there was no generalised enlargment of the kidney. All other cases were regarded as " severe ". No unaffected kidneys were encountered.

In addition to the condition of chronic nephritis, which was common to all groups, all the kidneys from rats treated with xanthopterin contained crystalline deposits which were thought to be xanthopterin itself. These deposits were both within and between cells of the renal tubules. Their presence appeared to give rise to no inflammatory response and had no obvious effect either on the severity of the chronic nephritis or on the induction of renal tumours.

### *Incidence of non-renal tumours*

This is shown in Table IV. Exposure to lead was not obviously associated with the development of tumours other than of the kidney.

### DISCUSSION

Clearly the results of the experiment were essentially negative. Xanthopterin, in doses sufficient to give rise to crystalline deposits in the kidneys, did not

TABLE IV.—*Primary Non-renal Neoplasms*

| Group | Treatment | No. of rats in group | No. of rats with non-renal neoplasms | Details of neoplasms | Day of death of rats with neoplasms |
|---|---|---|---|---|---|
| A | $L^{+++}$ | 24 | 0 | | |
| B | $L^{++}$ | 24 | 1 | Multiple papillomata and transitional-cell carcinoma of bladder | 441 |
| C | $L^{+}$ | 24 | 4 | 1—Adenocarcinoma of pancreas | 510 |
| | | | | 3—Localized lymphocytic neoplasm (all arising in lung) | 523, 532 and 566 |
| D | $L^{+}T$ | 16 | 3 | 1—Anaplastic carcinoma of prostate | 320 |
| | | | | 1—Haemangioma of mesenteric lymph-node | 483 |
| | | | | 1—Myxomatous tumour of body wall | 631 |
| E | $L^{+}X$ | 16 | 2 | 1—Spindle-cell sarcoma arising in pelvis | 225 |
| | | | | 1—Undifferentiated malignant tumour arising in neck | 274 |
| F | $L^{++}T$ | 16 | 3 | 1—Adenoma of lung | 99 |
| | | | | 1—Subcutaneous pleomorphic sarcoma | 523 |
| | | | | 1—Localized lymphocytic neoplasm arising in lung | 631 |
| G | $L^{++}X$ | 16 | 1 | Multiple papillomata of bladder | 434 |
| H | X | 16 | 0 | | |
| J | T | 24 | 2 | 1—Lymphosarcoma arising in wall of descending colon with metastases in mesenteric nodes | 509 |
| | | | | 1—Large subcutaneous fibroma | 631 |
| K | None | 24 | 4 | 1—Localized lymphocytic neoplasm arising in wall of caecum | 357 |
| | | | | 2—Malignant lymphoma arising in thymus | 510 and 615 |
| | | | | 1—Exocrine adenoma of the pancreas | 540 |

increase or decrease the incidence of renal tumours attributable to lead ; and testosterone in high dose was similarly without effect.

Since chronic nephritis was present in all the rats in the experiment it is not possible to say whether its presence was necessary for tumour induction. However, this seems unlikely in so far as the severity of the nephritic condition bore no relation to the tumour response : kidneys showing severe nephritis had no tumours, despite exposure to lead, whilst tumours were present in kidneys only slightly affected by chronic nephritis. van Esch *et al.* (1962) in discussing the relationship between chronic nephritis and renal tumours, implied that lead administration gives rise to both lesions. Unfortunately, they do not give details of the occurrence of chronic nephritis in their control animals.

It is interesting that, at the lowest level of administration, lead phosphate injections gave rise to no renal neoplasms (Groups C, D and E). This suggests that, for practical purposes anyway, there is a threshold dose level (between 29 and 145 mg. lead phosphate per rat in the present experiment) below which renal tumours are not induced.

In a follow-up study of 425 pensioners who had been previously exposed to lead in an accumulator factory Dingwall-Fordyce and Lane (1963) found " no evidence to suggest that malignant disease was associated with lead absorption ". There was an increase in the incidence of cerebrovascular catastrophies. The present experiments show that a large dose of lead is necessary to induce kidney tumours in rats. The amount of lead absorbed by the lead-workers covered in Dingwall-Fordyce and Lane's survey might well have been too low to be carcinogenic.

### SUMMARY

1. Three groups of 24 male CB Wistar rats were injected with lead phosphate repeatedly over a period of 34 weeks. The first group received a total dose of 450 mg. per rat; the second group, 145 mg. per rat; and the third, 29 mg. per rat. More than half the rats in the first two groups which survived for more than 200 days developed benign or malignant kidney tumours. No renal tumours developed in the third group.

2. Repeated injections of testosterone propionate or of xanthopterin failed to influence the induction of renal tumours by lead.

3. All the rats in the experiment, including untreated controls, suffered mildly or severely from chronic nephritis. The administration of lead, xanthopterin or testosterone had no obvious effect on the severity of the renal disease, and the severity of the disease was not obviously associated with tumour development.

4. Primary neoplasms of sites other than the kidney arose in approximately equal frequency in all groups, and there was no indication that any of the chemical agents influenced their occurrence.

We are most grateful to Miss Marjorie Butt, Mr. E. A. Sykes and Mrs. Kay Foster for help in the preparation of the manuscript, and should like to express our thanks to Miss Anne Walsh, Mrs. J. Merryweather and Mr. George Munro for skilled technical assistance with the experimental work.

This investigation has been supported by grants to the Chester Beatty Institute (Institute of Cancer Research: Royal Cancer Hospital) from the Medical Research Council and the British Empire Campaign for Research, and by the Public Health Service Research Grant No. CA-03188-08 from the National Cancer Institute, U.S. Public Health Service.

### REFERENCES

BOYLAND, E., DUKES, C. E., GROVER, P. L. AND MITCHLEY, B. C. V.—(1962) *Br. J. Cancer*, **16**, 283.

DINGWALL-FORDYCE, I. AND LANE, R. E.—(1963) *Br. J. ind. Med.*, **20**, 313.

VAN ESCH, G. J., VAN GENDEREN, H. AND VINK, H. H.—(1962) *Br. J. Cancer*, **16**, 289

FAIRHALL, L. T. AND MILLER, J. W.-(1941) *Publ. Hlth Rep., Wash.*, **56**, 1610.

HADDOW, A.—(1954) Ciba Foundation Symposium on 'Chemistry and Biology of Pteridines', edited by G. E. W. Wolstenholme and M. P. Cameron, London (Churchill) p. 100.

KLOPP, C., YOUNG, N. F. AND TAYLOR, H. C.—(1945) *J. clin. Invest.*, **24**, 189.

KORENCHEVSKY, V. AND HALL, K.—(1939) *Br. med. J.*, i, 4.

MATTHEWS, J. J. AND WALPOLE, A. L.—(1958) *Br. J. Cancer*, **12**, 234.

ROE, F. J. C. AND MITCHLEY, B. C. V.—(1962) *Rep. Br. Emp. Cancer Campn.*, **40**, 24.

SELYE, H.—(1939) *J. Urol.*, **42**, 637.

THORN, G. W. AND ENGEL, L. L.—(1938) *J. exp. Med.*, **68**, 299.

TÖNZ, O.—(1957) *Z. ges. exp. Med.*, **128**, 361.

ZOLLINGER, H. V.—(1953) *Virchows Arch. path. Anat. Physiol.*, **323**, 694.